# Supplementary material for: Photobactericidal activity activated by thiolated gold nanoclusters at low flux levels of white light
Source: Nat Commun. 2020 Mar 5;11:1207. doi: 10.1038/s41467-020-15004-6 (PMC7057968; doi:10.1038/s41467-020-15004-6)
Supplement: Supplementary file 1 — Supplementary Information [file 41467_2020_15004_MOESM1_ESM.pdf]

Supplementary information

Photobactericidal activity activated by thiolated gold nanoclusters at low flux levels of white light

Gi Byoung Hwang[a], He Huang[b], Gaowei Wu[b], Juhun Shin[a], Andreas Kafizas[c], Kersti Karu[a], Hendrik Du Toit[b], Abdullah M. Alotaibi[a], Layla Mohammad-Hadi[e], Elaine Allan[d], Alexander J. MacRobert[e], Asterios Gavriilidis[b], and Ivan P. Parkin[a]*

[a]*Materials Chemistry Research Centre, Department of Chemistry, University College London, 20 Gordon Street, London, WC1H 0AJ, United Kingdom*

[b]*Department of Chemical Engineering, University College London, Torrington Place, London, WC1E 7JE, United Kingdom*

[c]Grantham Institute, Imperial College London, Exhibition Road, London, SW7 2AZ, *United Kingdom*

[d]*Department of Microbial Diseases, UCL Eastman Dental Institute, University College London, 256 Grays Inn Road, London, WC1X 8LD, United Kingdom*

[e]*UCL Division of Surgery and Interventional Science, Royal Free Campus, Rowland Hill Street, London, NW3 2PF, United Kingdom*

[*] To whom correspondence should be addressed.

E-mail: i.p.parkin@ucl.ac.uk Tel: 44(0)207 679 4669

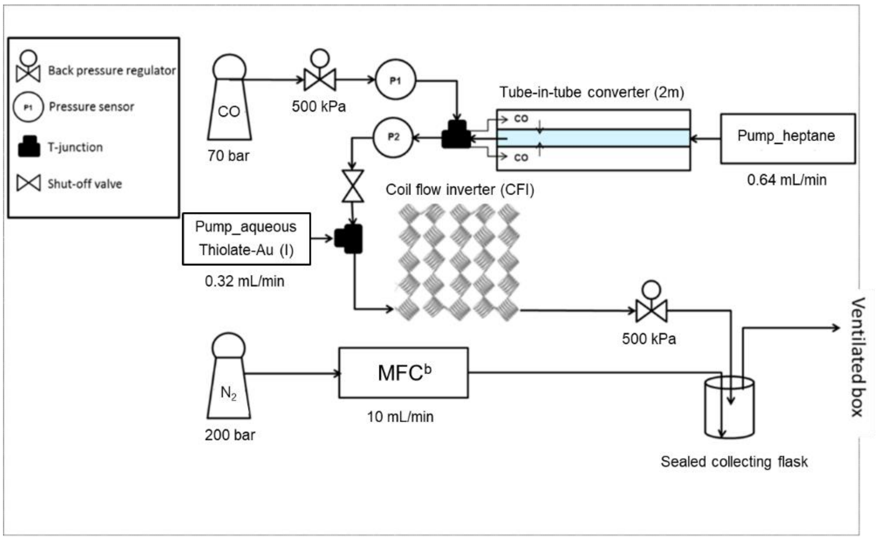

Supplementary Figure 1 | Set-up of microfluidic segmented flow system to synthesize [$Au_{25}(Cys)_{18}$] [b] MFC is Mass flow controller

The isotope patterns of peaks # 2 – #7 in Figure 1c are shown in Supplementary Figure 2. The theoretical isotope patterns of the peaks were simulated based on input empirical formulations shown in Supplementary Table 1 using Thermo Xcalibur software. The theoretical isotope patterns predicted by the software perfectly matched the isotope pattern from the experiment, identifying [$Au_{25}(Cys)_{18}$].

Supplementary Table 1 Empirical formulation and charge for theoretical simulation

Peak number	Empirical formulation	Charge
#1	Au_{25} C_{54} H_{105} N_{18} O_{36} S_{18}	3-
#2	Au_{25} C_{54} H_{104} N_{18} O_{36} S_{18} Na_{1}	3-
#3	Au_{25} C_{54} H_{103} N_{18} O_{36} S_{18} Na_{2}	3-
#4	Au_{25} C_{54} H_{102} N_{18} O_{36} S_{18} Na_{3}	3-
#5	Au_{25} C_{54} H_{101} N_{18} O_{36} S_{18} Na_{4}	3-
#6	Au_{25} C_{54} H^{100} N_{18} O_{36} S_{18} Na_{5}	3-
#7	Au_{25} C_{54} H_{99} N_{18} O_{36} S_{18} Na_{6}	3-

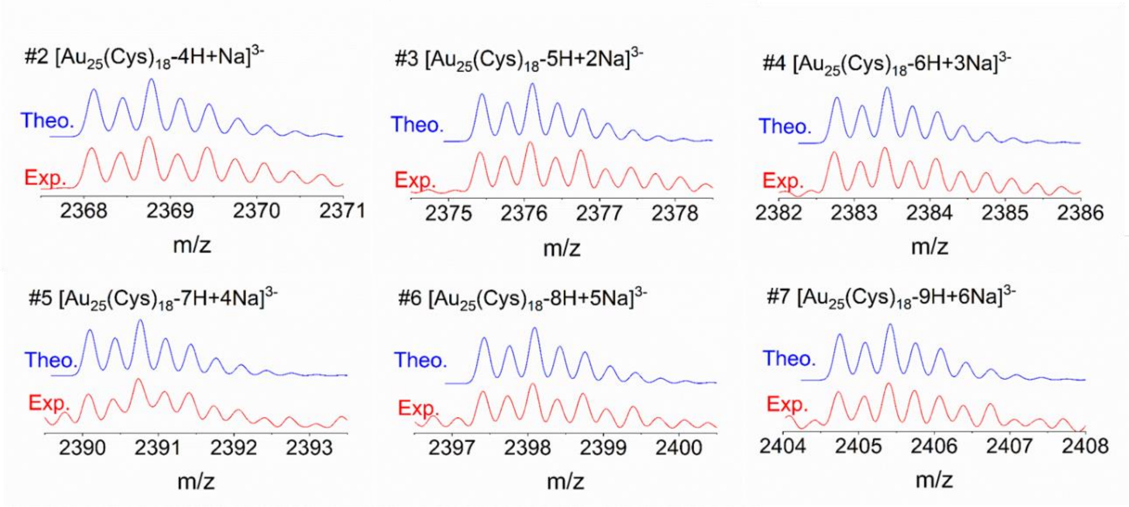

Supplementary Figure 2 | Theoretical and experimental isotope patterns of peaks #2-#7 in Figure 1c.

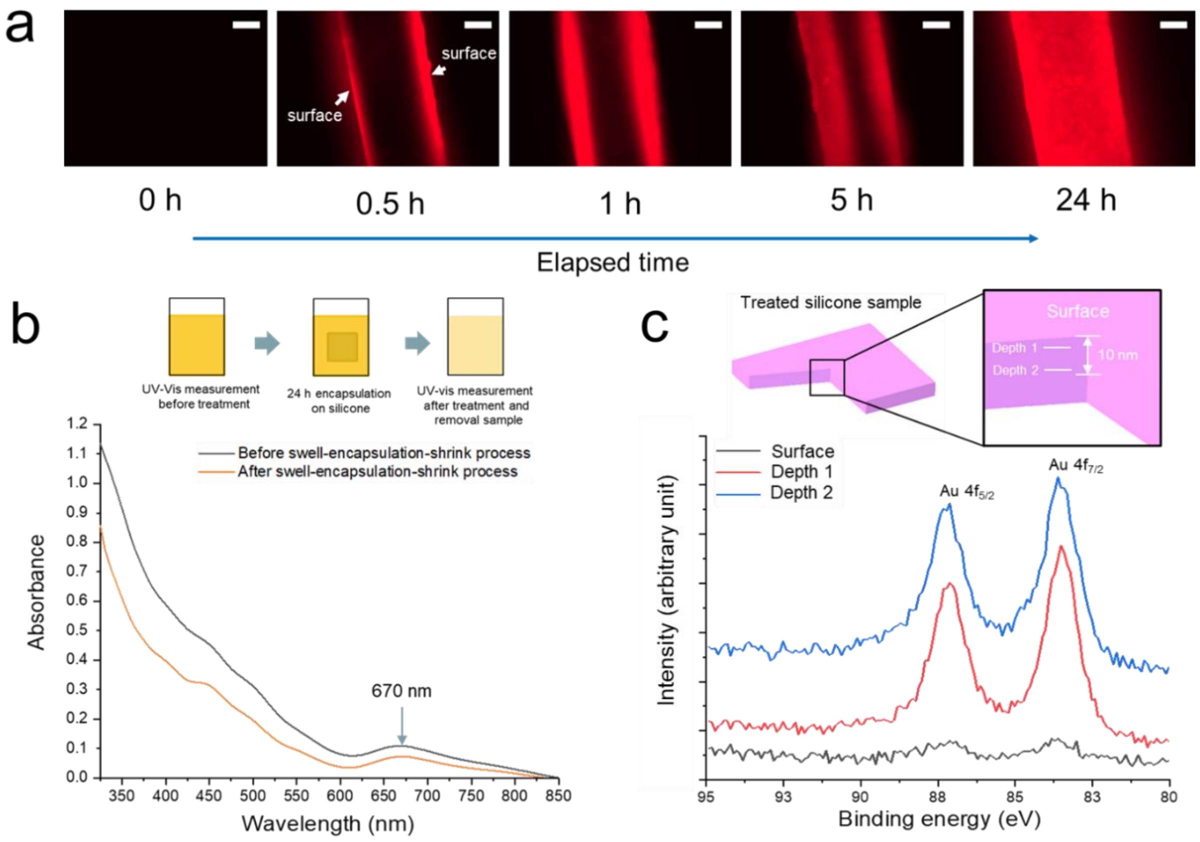

Supplementary Figure 3| Characterization of crystal violet and [$Au_{25}(Cys)_{18}$] in silicone. a, Photoluminescence of crystal violet inside silicone after 0.5, 1, 5 and 24 h encapsulation (λ_{Ex} = 545 nm, λ_{Em} = 620 nm, the scale bar length = 50 μm). **b**, UV-Vis spectra of [$Au_{25}(Cys)_{18}$] solution before and after swell-encapsulation-shrink, **c,** XPS spectra of [$Au_{25}(Cys)_{18}$] only encapsulated silicone sample.

A swell-encapsulation-shrink process was employed to produce bactericidal silicone. In the swell-encapsulation process, CV molecules and [$Au_{25}(Cys)_{18}$] clusters penetrated into the polymer matrix as the silicone was swelled by acetone containing the reagents, and then when the polymer was removed from the solution, it shrank and CV and [$Au_{25}(Cys)_{18}$] remained inside the polymer. The photobactericidal silicone was prepared through a 24 h process. The polymer samples with various encapsulation times were analysed using fluorescence microscopy. CV fluorescence of thinly sliced sample side section was photographed and then the image was analysed to determine CV diffusion throughout the sample. Supplementary Figure 3a shows the CV diffusion inside the silicone with increasing encapsualtion time. The images show that the gradient of CV diffusion across the sliced polymer. At the beginning of encapsulation, CV penetration was predominantly near the surface of the sample and then the CV diffused throughout the polymer with increasing time until it was saturated after 24 h. The amount of [$Au_{25}(Cys)_{18}$] encapsulated into silicone was measured before and after the encapsulation process by measuring absorbance at 670 nm. The number of [$Au_{25}(Cys)_{18}$] clusters in the mixture was about 7.2×10^{15} [$Au_{25}(Cys)_{18}$] clusters mL^{-1}. As shown in Supplementary Figure 3b, it was observed that after the swell-encapsulation-shrink, about 33 % of the [$Au_{25}(Cys)_{18}$] clusters were encapsulated into the silicone samples indicating that approximately 2.4×10^{15} [$Au_{25}(Cys)_{18}$] clusters mL^{-1} penetrated into the polymer. XPS sputter depth profiling was employed to determine the existence of [$Au_{25}(Cys)_{18}$] in the silicone surface and within the polymer. As shown in Supplementary Figure 3c, a doublet peak at 87 and 83 eV corresponding to gold $4f_{5/2}$ and $4f_{7/2}$ was observed at the surface and within the polymer bulk. This indicates that [$Au_{25}(Cys)_{18}$] clusters were successfully encapsulated on the polymer surface and within the silicone matrix.

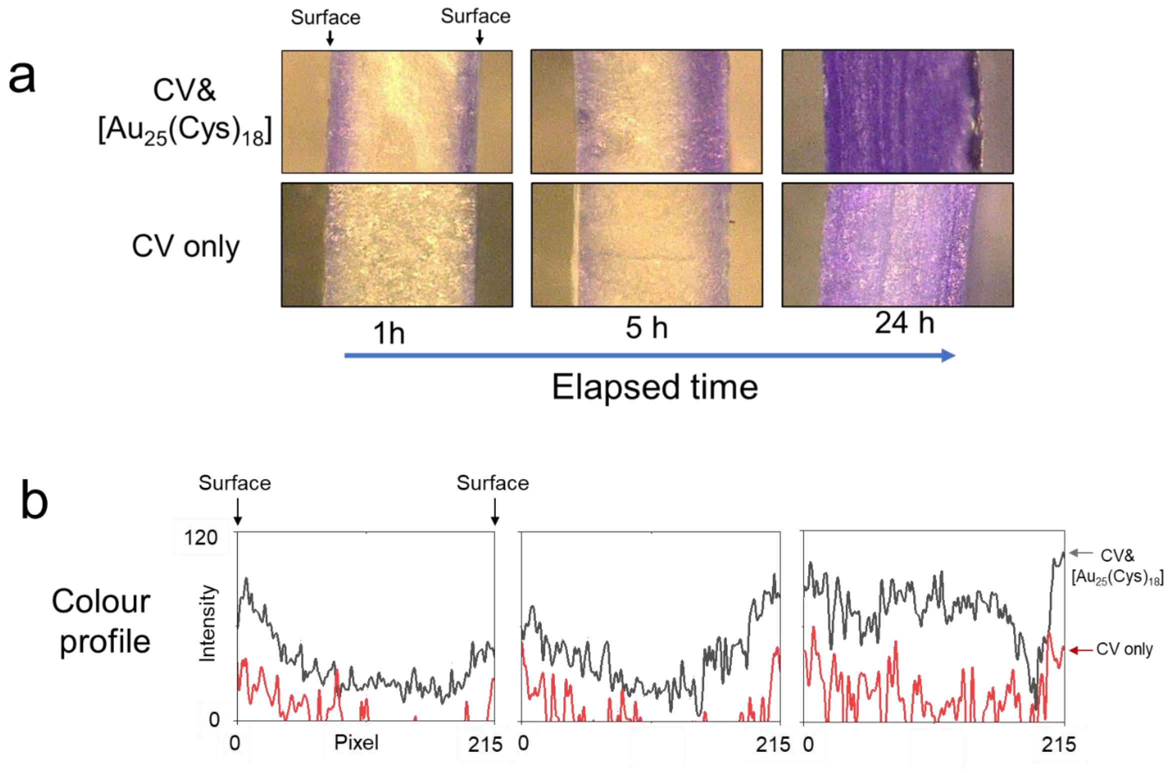

Supplementary Figure 4 Distribution of crystal violet in CV only and CV&[$Au_{25}(Cys)_{18}$] polymer. a, Side section image of sliced CV only and CV&[$Au_{25}(Cys)_{18}$] polymers after 1, 5 and 24 h encapsulation. **b**, Profile of CV distribution inside CV only and CV&[$Au_{25}(Cys)_{18}$] polymers after 1, 5 and 24 h encapsulation. CV indicates crystal violet

The side images of thinly sliced CV only and CV&[$Au_{25}(Cys)_{18}$] polymers were taken by optical microscope and they were analysed using ImageJ. As shown in Supplementary Figure 4, CV diffusion into the silicone was enhanced after additional encapsulation of [$Au_{25}(Cys)_{18}$] and after 24 h encapsulation, the polymer containing [$Au_{25}(Cys)_{18}$] exhibited more intense violet colour indicating that silicone with CV&[$Au_{25}(Cys)_{18}$] has more CV molecules inside the polymer than the polymer with CV alone.

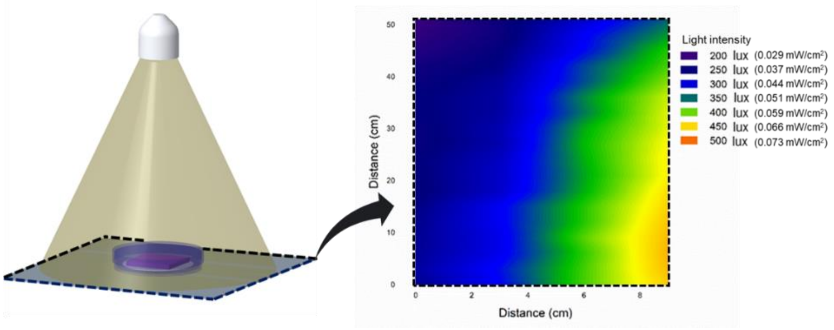

Supplementary Figure 5 |White light source. Intensity distribution of white light used for irradiation of treated samples. Colour scale correspond from 200 (purple) to 500 lux (orange).

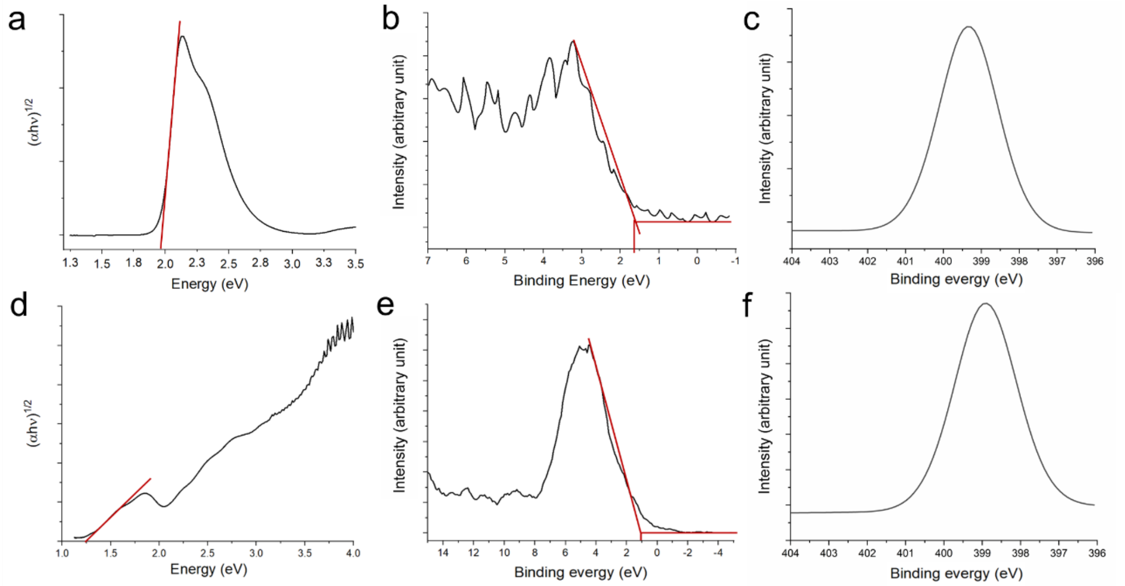

Supplementary Figure 6 | Band gap, homo band and N 1s spectra from phase-pure crystal violet and [Au_{25}(Cys)$_{18}$]. a, band gap, **b**, homo band and **c**, N 1s spectrum for phase-pure crystal violet. **d**, band gap, **e**, homo band and **f**, N 1s spectrum for phase pure [Au_{25}(Cys)$_{18}$].

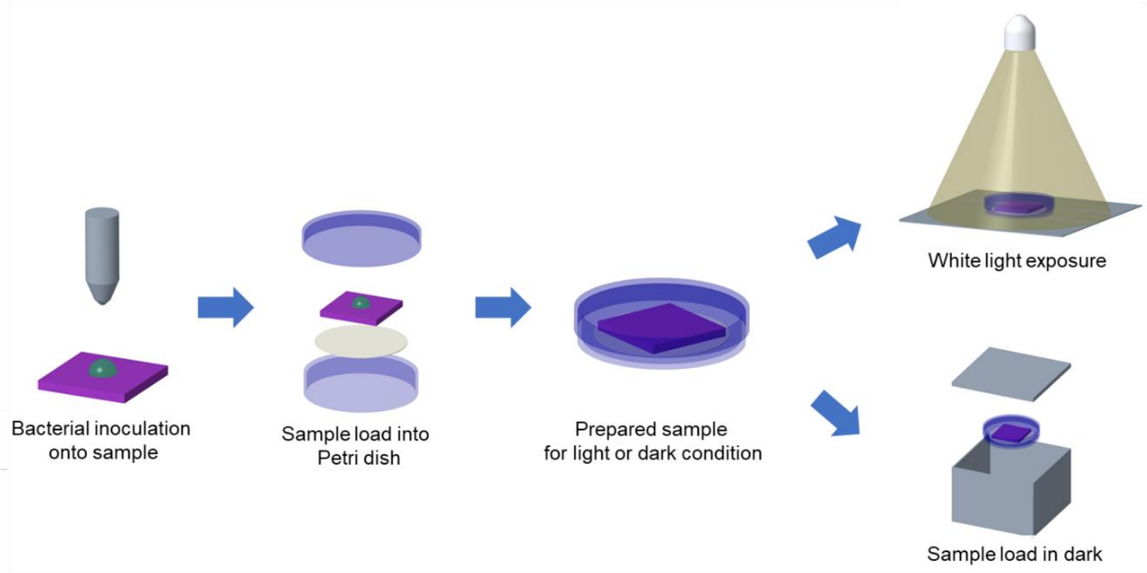

Supplementary Figure 7 | Procedure of bactericidal test
